# Fabrication and Evaluation of Graphene Oxide/Hydroxypropyl Cellulose/Chitosan Hybrid Aerogel for 5-Fluorouracil Release

**DOI:** 10.3390/gels8100649

**Published:** 2022-10-12

**Authors:** Yanan Sang, Pengpai Miao, Tao Chen, Yuan Zhao, Linfeng Chen, Yayang Tian, Xiaobing Han, Jie Gao

**Affiliations:** 1 School of Pharmacy, Hubei University of Science and Technology, Xianning 437100, China; 2 Hubei Key Laboratory of Radiation Chemistry and Functional Materials, Non-Power Nuclear Technology Collaborative Innovation Center, Hubei University of Science and Technology, Xianning 437100, China

**Keywords:** graphene oxide, hydroxypropyl cellulose, chitosan, hybrid aerogel, drug release

## Abstract

The incorporation of graphene oxide (GO) into a polymeric drug carrier can not only enhance the loading efficiency but also reduce the initial burst and consequently improve the controllability of drug release. Firstly, 5-fluorouracil (5-Fu)-loaded hydroxypropyl cellulose/chitosan (HPC/CS@5-Fu) and GO/HPC/CS@5-Fu aerogels were successfully fabricated through chemical cross-linking with glutaraldehyde. Then, the obtained aerogels were characterized using scanning electron microscopy (SEM), Fourier transform infrared (FITR), X-ray diffraction (XRD), differential scanning calorimetry (DSC), thermogravimetry (TG), and the effect of HPC and GO content on the drug loading (DL) and encapsulation efficiency (EE) for the two aerogels were investigated, respectively. Finally, the drug release behavior of the GO/HPC/CS@5-Fu aerogels with different GO content was evaluated at two different pH values, and four kinds of kinetic models were used to evaluate the release behavior.

## 1. Introduction

Cancer is one of the most serious diseases around the world, millions of people are diagnosed with cancer annually. What is more, as the result of the adoption of unhealthy habits and population aging, the trends of cancer cases are increasing [1,2]. In the last two decades, 5-fluorouracil (5-Fu) has become one of the most effective chemotherapeutic drugs for the treatment of cancer [3,4]. Although 5-Fu is an effective chemotherapy drugs, poor efficiency and high dosage requirements are still observed in cancer treatment, which can be ascribed to the low loading efficiency and initial burst of the drug carrier. Therefore, the development of a high-performance carrier with high loading efficiency and reduced initial burst for cancer treatment is highly desirable [5].

Gels (including hydrogel and aerogel) consist of a hydrophilic polymer with a network structure, which can obviously swell but is insoluble in water. Because of its hydrophilicity, biocompatibility, biodegradability, and drug loading ability, gel was widely used in the field of drug delivery, self-healing, and water retention [6,7]. Due to the extensive sources and low cost, biodegradable, natural, polysaccharide-based gel was widely used as carrier in drug delivery [8,9,10].

Among the natural polysaccharides, chitosan (CS) has attracted much attention in medical fields [11,12] and is widely used for the loading and release of 5-Fu. CS-decorated nanoemulsion gel used for the topical delivery of 5-Fu was reported by the group of Lim [13]. The incorporation of a nanoemulsion into CS gel extended the half-life and enhanced the skin’s drug retention, which is suitable for the treatment of melanoma. CS nanoparticles were synthesized through ionic gelation and used for the pH-stimulated delivery of 5-Fu. In vitro release results indicated a controlled and sustained release of 5-Fu, with release amounts in the range of 29.1–60.8% [14]. Cross-linked CS microspheres was prepared for the colonic delivery of 5-Fu; these CS microspheres can release 5-Fu in both strong acidic (pH = 1.2, 40%) and weak basic (pH = 7.4, 60%) conditions [15]. CS-based polymeric composites was also reported for the release of 5-Fu. CS/methylcellulose nanospheres were reported for the loading of 5-Fu, and the release process was found to follow the Fickian mechanism [16]. In addition, CS/hydroxypropylmethyl cellulose was also used in the delivery of 5-Fu, and controlled release behavior was observed for the blended microspheres for up to 10 h [17].

To further enhance the loading efficiency and reduced the initial burst of the CS-based carrier, aromatic graphene oxide (GO) was incorporated into the construction of the drug carrier [18,19,20]. The huge specific surface area of GO is capable of immobilizing drugs with π–π stacking and hydrogen bond, which has an important effect on the drug loading and release [21,22,23,24]. CS/GO bionanocomposite beads were developed for the loading of metronidazole; the obtained carrier has good potential to minimize the multiple-dose frequency with its sustained drug release property and can reduce the side effects [25]. CS/GO microspheres have also been used for the pH-controlled release of amoxicillin; the drug loading efficiency was notably enhanced after the incorporation of GO, and 40.22% and 15.18% of drug release was observed at pH 1.2 and pH 7.4, respectively [26]. The CS/GO system was also used for the release of 5-Fu; Ha and co-workers reported the synthesis and 5-Fu delivery behavior of CS-modified GO hybrid nanosheets [27]. 5-Fu was loaded successfully on CS-g-GO sheets, and controlled release behavior and long-term biocompatibility were observed for this carrier. Recently, a CS/carboxymethyl cellulose/GO hybrid aerogel was prepared by the ion cross-linking of calcium and used for the loading of 5-Fu [28]. The release of 5-Fu can be controlled by the pH value, and the kinetic process follows Fickian diffusion.

The incorporation of GO has a great impact on the behavior of 5-Fu loading and release; however, there are few reports about the effect of GO content for these polysaccharide carriers. Therefore, a covalent cross-linking aerogel based on CS, hydroxypropyl cellulose (HPC), and GO was synthesized for the delivery of 5-Fu. The effect of the GO content on the drug loading efficiency and the release behavior were investigated in detail. In addition, the release kinetics at two different pH values were also evaluated with four different models.

## 2. Results and Discussion

### 2.1. Characterization of Aerogel

#### 2.1.1. Morphology Analysis

**Figure 1:**
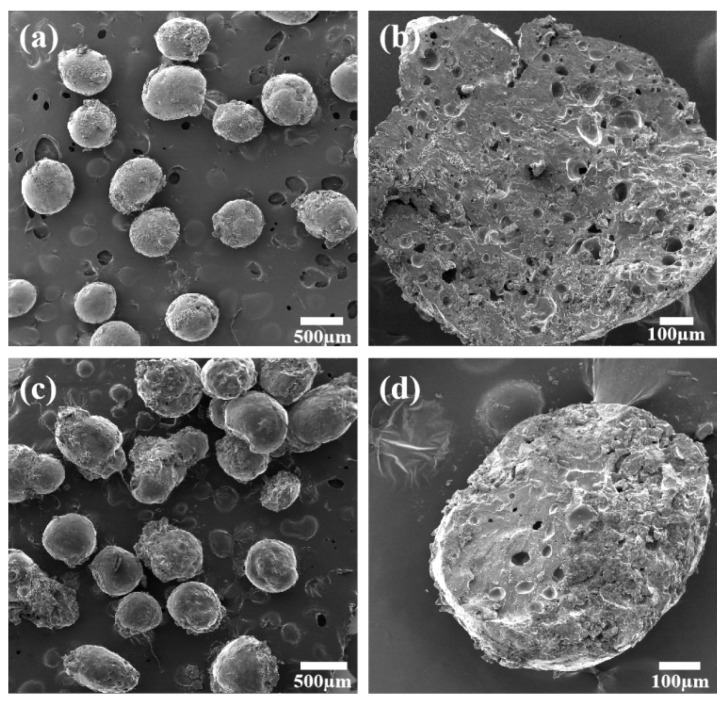
SEM images of HPC/CS@5-Fu (3:2) (**a**) surface and (**b**) cross section, GO/HPC/CS@5-Fu (0.1:3:2) (**c**) surface and (**d**) cross section.

The surface and cross-section morphology of HPC/CS@5-Fu and GO/HPC/CS@5-Fu aerogels were investigated using SEM techniques and are illustrated in Figure 1. As shown Figure 1a, the HPC/CS@5-Fu clearly depicts a uniform spherical shape and has diameters in the range of 0.3 to 0.6 mm. In addition, the surface appears smooth and regular, which is consistent with previous work [12,17]. With the incorporation of GO (Figure 1c), the surface became rough and irregular, which can be ascribed to the GO sheet’s wrinkled structure, leading to a loss of the spherical shape. The cross section of the HPC/CS@5-Fu aerogel exhibits a porous structure, while the cross section of GO/HPC/CS@5-Fu is denser with very few visible cavities. This is due to the enhanced cross-linking density of glutaraldehyde with the hydroxyl group originating from GO [25,26], and the difference in the porous structure of HPC/CS@5-Fu and GO/HPC/CS@5-Fu will have an influence on the release of 5-Fu.

#### 2.1.2. Composition Analysis

**Figure 2:**
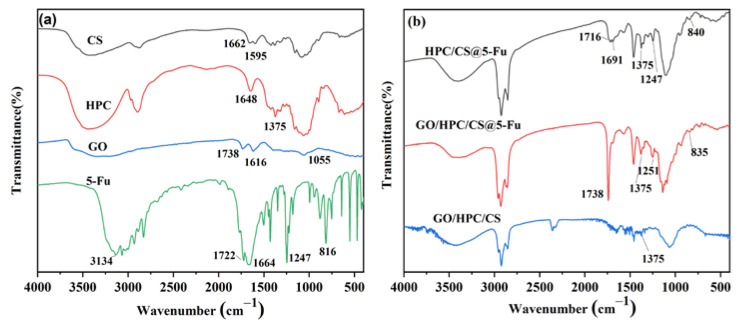
FTIR spectra of (**a**) raw material and (**b**) aerogels.

The FTIR spectra of raw material such as CS, HPC, GO, 5-Fu, and the obtained aerogels were obtained to assign the functional groups and the interaction between them (Figure 2). As show in Figure 2a, a broad peak from 3300 to 3500 cm^−1^ of -OH and -NH_2_ groups was observed for CS; C=O stretching of amide I at 1662 cm^−1^ and N-H bending of amide II at 1595 cm^−1^ were also observed [25]. For the spectrum of HPC, the C-H stretching for -CH_2_- and C-O stretching were observed at 2895 and 1064 cm^−1^, respectively [23]. For the spectrum of GO, the bands at 3400 cm^−1^, 1738 cm^−1^, and 1055 cm^−1^ were clearly observed for the O-H stretching, C=O stretching, and C-O stretching, respectively [29]. The FTIR spectrum of 5-Fu clearly marked the presence of 5-Fu as evident from the observed bands at 3134 cm^−1^ for C=C stretching of aromatic group, 1664 cm^−1^ for C=O stretching, 1247 cm^−1^ for C-F stretching, and 816 cm^−1^ for C-H bending in -CF=CH- [28,30]. As shown in Figure 2b, all the vibration peaks for the corresponding raw materials were observed in the HPC/CS@5-Fu and GO/HPC/CS@5-Fu aerogels, which revealed the successful loading of 5-Fu. In addition, a new peak for imines (C=N) at 1691 cm^−1^ was observed, which demonstrated the cross-linking of CS, HPC, and GO with glutaraldehyde [26,31,32].

#### 2.1.3. X-ray Powder Diffraction Analysis

**Figure 3:**
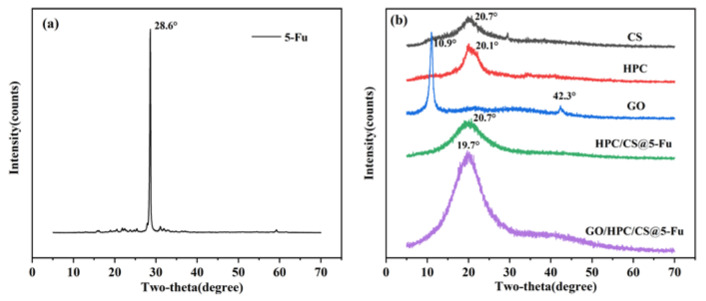
XRD patterns of (**a**) 5-Fu and (**b**) CS, HPC, GO, and aerogels.

The XRD patterns of the raw materials and aerogels are shown in Figure 3. The XRD pattern of pure 5-Fu is shown in Figure 3a, which contains a sharp peak at 28.6°, suggesting the crystalline nature of 5-Fu. This peak disappeared in 5-Fu-loaded aerogel, which indicated the uniform molecular dispersion of 5-Fu in the polymer matrix [15,30]. CS and HPC show a broad peak at 20.7° and 20.1° (Figure 3b), respectively, corresponding to its backbone, which is consistent with previous work [15,23]. The XRD pattern of GO contains two characteristic peaks at 10.9° and 42.3° corresponding to the (001) and (100) planes, demonstrating the full exfoliation of graphite oxide into GO [19,25]. For the XRD patterns of the two drug-loaded aerogels, only the characteristics of CS and HPC at 20.7° and 19.7° were observed, respectively, which reveal the formation of the desired aerogels [15,23,26].

#### 2.1.4. Differential Scanning Calorimeter Analysis

**Figure 4:**
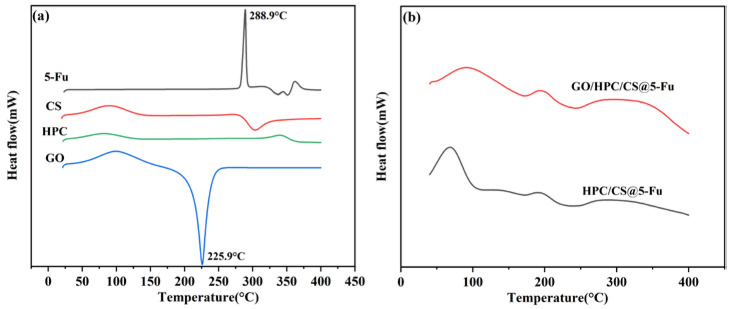
DSC curves of (**a**) raw material and (**b**) aerogels.

The DSC curves of the raw materials and aerogels are shown in Figure 4. 5-Fu shows a sharp endothermic peak at 288.9 °C, which can be ascribed to the melting point of 5-Fu. This characteristic peak was not observed in the drug-loaded aerogel, demonstrating that the 5-Fu was molecularly well dispersed again [16,17]. The CS and HPC show a Tg around 118 and 102 °C, respectively, corresponding to the movement of the chain segment [12,16], and a sharp exothermic peak at 303 °C was observed for the decomposition of CS [15]. The DSC curves of GO suggested that the decomposed started from 180 °C, and the oxygen-containing groups were completely destroyed at 225.9 °C [19]. In the case of 5-Fu-loaded aerogels, a strong peak was observed at 70 and 90 °C for HPC/CS@5-Fu and GO/HPC/CS@5-Fu, respectively. In addition, a small peak at 190 °C and a broad peak at 300 °C were observed due to endothermic transitions, which is consistent with previous work [12,15].

#### 2.1.5. Thermogravimetry Analysis

**Figure 5:**
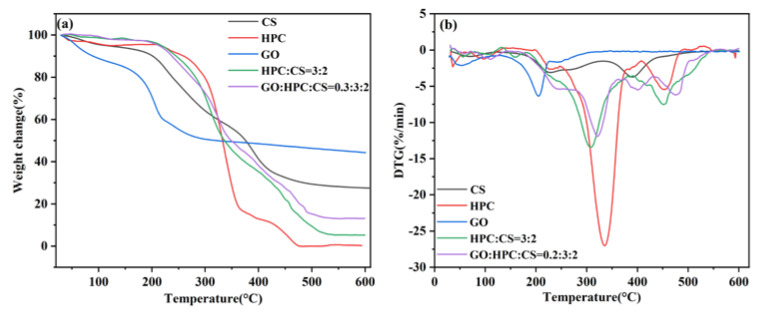
TG (**a**) and DTG (**b**) curves of CS, HPC, GO, and aerogels.

The TG and DTG curves of the raw materials and aerogels are shown in Figure 5. Compared with the HPC/CS@5-Fu and GO/HPC/CS@5-Fu aerogels, all of the raw materials show weight loss before 150 °C, which can be ascribed to the loss of combined water (bound to hydrophilic groups via hydrogen bonding) [9]. The CS shows two degradation stages, the temperatures of maximum weight loss rate (*T_mr_*) are 235 and 387 °C, with a high residue of 27.6% at 600 °C [15]. The HPC shows multiple degradation stages, the *T_mr_* is 336 °C, and it was almost completely decomposed at 600 °C [9]. The GO shows fast weight loss at 203 °C, corresponding to the loss of oxygen-containing groups, with the highest residue of 44.4% at 600 °C [20]. For the two aerogels, similar degradation behaviors were observed, and the *T_mr_* increased from 310 to 320 °C with the incorporation of GO [20,29,33]. The maximum weight loss rate for the two aerogels was comparatively slower than that of pure HPC, indicating that the obtained aerogels possess better thermal stability [9].

### 2.2. Drug Loading (DL) and Encapsulation Efficiency (EE) of Aerogel

**Figure 6:**
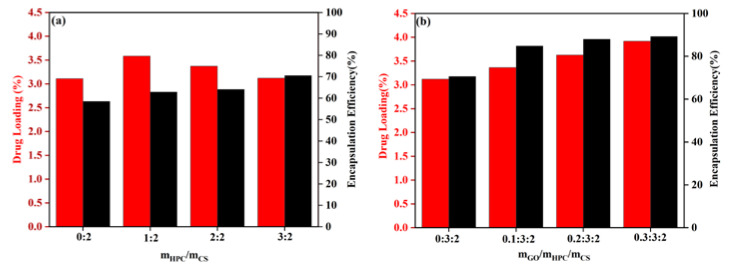
DL and EE of (**a**) HPC/CS@5-Fu and (**b**) GO/HPC/CS@5-Fu.

The effects of HPC and GO content on the DL and EE for HPC/CS@5-Fu and GO/HPC/CS@5-Fu are shown in Figure 6. As shown in Figure 6a, with the increase of HPC content, the DL firstly increased and then decreased; the highest DL was 3.58%. The EE increased with the increase of HPC content; the highest EE (70.56%) was obtained for m_HPC_/m_CS_ = 3:2, which can be ascribed to more hydrogen bonds being formed [8]. As shown in Figure 6b, with the increase of GO content, both the DL and EE increased obviously, which will reduce the number of doses. The highest DL and EE were 3.91% and 89.24%, which can be ascribed to the increase of hydrogen bonds and π–π stacking between 5-Fu and GO [25,26]. 

### 2.3. In Vitro Release of 5-Fu from Aerogel

#### 2.3.1. Release of 5-Fu under Different pH Values

**Figure 7:**
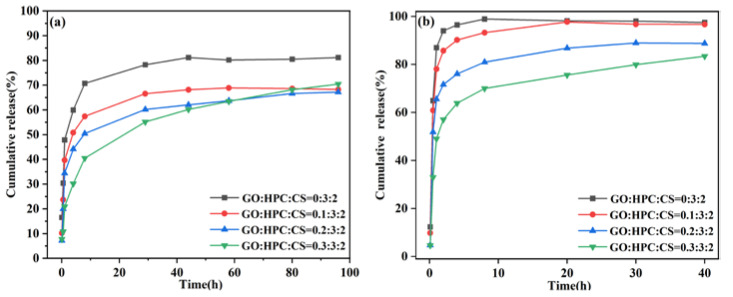
Cumulative release of 5-Fu under (**a**) pH = 7.4 and (**b**) pH = 5.0.

Based on the results obtained, GO/HPC/CS@5-Fu aerogels with different GO contents were used in drug release investigation. As shown in Figure 7, for the release of 5-Fu from aerogels without GO (0:3:2), an obvious initial burst was observed, especially at pH = 7.4. This will not only cause side effects but also leads to wastage of the drug [18]. At both pH conditions, a reduced initial burst was observed for all the aerogels containing GO. This can be ascribed to the strong π–π stacking between 5-Fu and GO, which can lower the rate of 5-Fu release, thus reducing side effects and enhancing drug utilization [19]. With the increase of GO content, the initial burst was gradually suppressed, and the aerogel exhibited sustained release behavior of 5-Fu. This is consistent with previous work, demonstrating that the GO is a good candidate for drug loading and release [25,26]. In addition, for the same drug-loaded aerogel, the pH value has a dramatic influence on the release behavior. The release of 5-Fu is much faster and complete under pH = 5.0 than at pH = 7.4, which is due to the presence of -NH_2_ groups in the aerogels. This structure easily expands in the acidic media, so the diffusion of drug occurred easily [26,27]. The pH sensitivity of 5-Fu release from aerogels will be useful for drug release in the targeted tumor regions [14,25].

#### 2.3.2. Release Kinetics of Different Aerogels

The release kinetics can reveal the release mechanism and determine the potential application of the obtained aerogel carrier. To give a deep insight into the release process, four kinetic models were used to evaluate the release behavior for different GO/HPC/CS@5-Fu aerogels, including zero-order, first-order, Higuchi, and Korsmeyer–Peppas models (Equations (1)–(4)) [9,14,15,26].

Zero-order model:

*M_t_/
M_∞_* = *K*_0_*t* (1)

First-order model:

ln (1 − *M_t_/M_∞_*) = −*K*_1_*t* (2)

Higuchi model:

*M_t_/M_∞_* = *K_H_t*^1/2^ (3)

Korsmeyer–Peppas model:

*M_t_/M_∞_ = K_KP_tn* (4)

where *K_0_* is the kinetic dissolution constant, *K*_1_ and *K_H_* is the kinetic constant, *K_KP_* is a proportional constant, *n* is the diffusional exponent.

**Figure 8:**
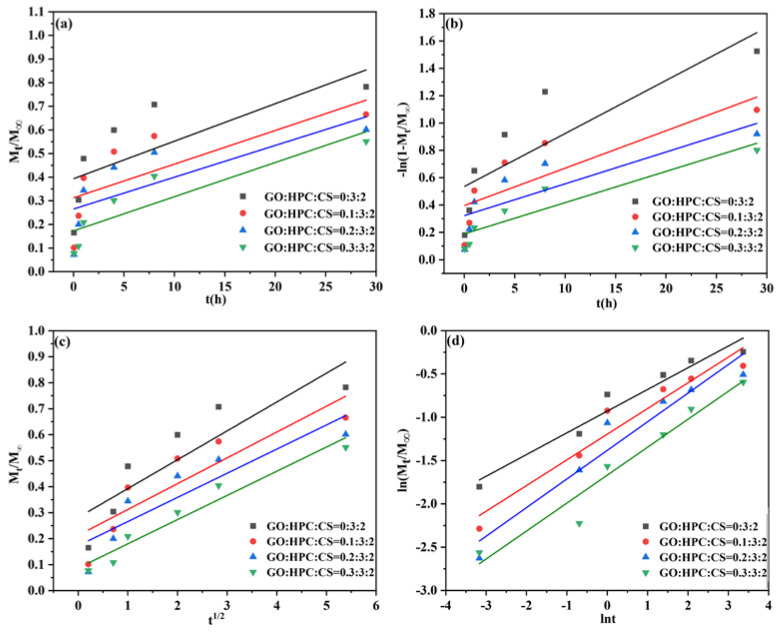
The linear fitted plots of the 5-Fu release from different aerogels with (**a**) zero-order, (**b**) first-order, (**c**) Higuchi, and (**d**) Korsmeyer–Peppas models at pH = 7.4.

**Figure 9:**
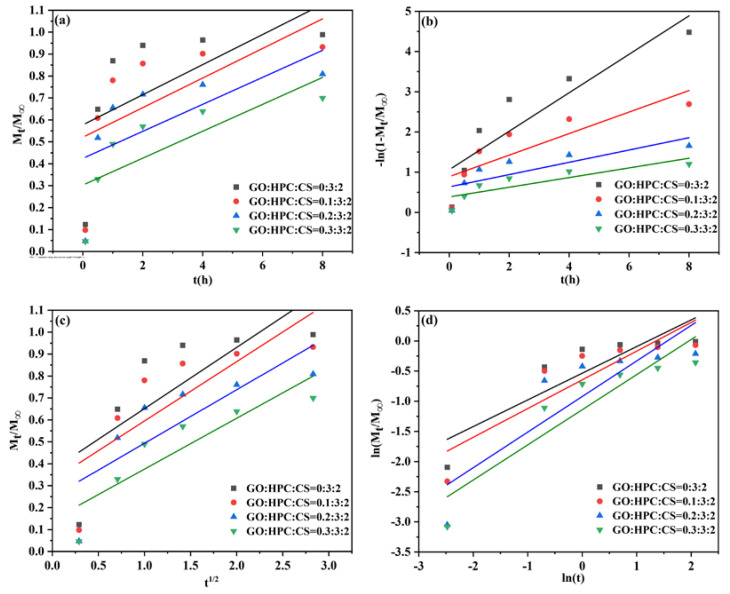
The linear fitted plots of the 5-Fu release from different aerogel with (**a**) zero-order, (**b**) first-order, (**c**) Higuchi, and (**d**) Korsmeyer–Peppas models at pH = 5.0.

The fitting curves of 5-Fu release from different GO/HPC/CS@5-Fu aerogels with the four kinetic models are shown in Figure 8 (pH = 7.4) and Figure 9 (pH = 7.4), the corresponding parameters and the coefficient (*R*^2^) calculated from these curves are listed in Table 1 and Table 2, respectively.

As seen from Table 1, according to the *R*^2^ value, release of 5-Fu from all aerogels with different GO contents fitted well to the Korsmeyer–Peppas model at pH = 7.4, indicating that 5-Fu release is controlled by diffusion [26]. In addition, the value of *n* is <0.5, revealing that the drug release process is controlled by Fickian diffusion [28].

As seen from Table 2, the release of 5-Fu at pH = 5.0 was also controlled by the Korsmeyer–Peppas model with non-Fickian diffusion, except for the HPC/CS@5-Fu aerogels. The highest *R*^2^ value of HPC/CS@5-Fu aerogel was observed for the first-order model, indicating that 5-Fu release is controlled by the initial burst [34]. With the incorporation of GO, the initial burst was obviously suppressed, again demonstrating that GO can improve the sustained release of 5-Fu and the drug utilization.

### 2.4. Comparison Study

Different investigations based on GO and CS biocomposites as drug carriers have been compared with the present study in Table 3. As seen in the table, relatively high drug loading was obtained for the fabricated CS/HPC/GO aerogel microspheres. This can be ascribed to the porous structure of CS/HPC/GO in this work, though it possesses a larger size than other nanospheres or nanoparticles. In addition, the release time of CS/HPC/GO is much longer than that of most of the present carriers, this is due to the strong interaction between the drug and GO [19,25,26], as well as the stronger chemical cross-linking of glutaraldehyde than that of calcium ion [16,28].

The fabricated GO/HPC/CS@5-Fu aerogel is microsized, which is not suitable for intravenous injection. On the other hand, it is sensitive to pH, which is not suitable for oral. According to the literature [13,21], GO/HPC/CS@5-Fu aerogel is suitable for skin implants or intratumoral injection. Future work will focus on skin drug retention and tumor growth inhibition testing after drug administration.

## 3. Conclusions

In summary, drug-loaded GO/HPC/CS@5-Fu aerogels with different GO contents were fabricated through emulsification and chemical cross-linking. The obtained aerogel had a spherical shape with micro-sized diameter; XRD and DSC demonstrated the uniform molecular dispersion of 5-Fu in the polymer matrix. The DL and EE of 5-Fu increased obviously with the increase of GO content, which will reduce the number of doses required. With the increase of GO content, the initial burst was dramatically suppressed, and the aerogel exhibited sustained release behavior of 5-Fu, which will enhance drug utilization. In addition, the obtained GO/HPC/CS@5-Fu aerogel is sensitive to pH value, which will be useful for drug release in the targeted tumor regions. Finally, the result of the kinetics investigation revealed that the release of 5-Fu fits the Korsmeyer–Peppas model. Fickian and non-Fickian diffusion were observed for the release at pH = 7.4 and pH = 5.0, respectively. These findings open a new approach for the construction of drug carriers based on natural polysaccharides for sustained and stimuli-sensitive drug release.

## 4. Materials and Methods

### 4.1. Materials

All reagents were purchased from suppliers of analytical reagents and used without purification. Graphene oxide (GO) was purchased from Suzhou TanFeng Graphene Tech Co., Ltd. (Suzhou, China). Chitosan (CS, deacetylation degree >95%), hydroxypropyl cellulose (HPC, Mw = 100,000), glacial acetic acid (99.5%), and glutaraldehyde (25% in H_2_O) were purchased from Sinopharm Chemical Reagent Co., Ltd. (Shanghai, China). 5-Fluorouracil (99%), Tween-80, liquid paraffin, and petroleum ether were supplied by Maclin Biochemical Technology Co., Ltd. (Shanghai, China). Sodium hydroxide, potassium chloride, potassium dihydrogen phosphate, sodium dihydrogen phosphate dihydrate, sodium dihydrogen phosphate dihydrate, trichloromethane, and hydrochloric acid were all purchased from HWRK chemical Co., Ltd. (Beijing, China). Distilled water was used throughout the experiments.

### 4.2. Preparation of HPC/CS@5-Fu Aerogel Microspheres

The 5-Fu-loaded composite aerogel microspheres were fabricated via emulsification and chemical cross-linking [17,26,31,32,33]. In a typical procedure, 0.3 g CS was dissolved into 15 mL 5% acetic acid aqueous solution, and 0, 0.15, 0.3, or 0.45 g HPC was added to the dispersion under stirring until complete dissolution at 50 °C. Then, 0.1 g 5-FU was added to the mixture and stirred for an hour; the obtained mixture was poured into an emulsifier dispersion, which consisted of 1.8 mL Tween-80, 49 mL petroleum, and 35 mL liquid paraffin. Half an hour later, 2 mL glutaraldehyde was dropped within 30 min, and the chemical cross-linking reaction was maintained at 60 °C for another hour. Finally, the fabricated HPC/CS@5-Fu aerogel was washed with hexane and water alternately and dried at room temperature for 24 h in a vacuum oven.

### 4.3. Preparation of GO/HPC/CS@5-Fu Aerogel Microspheres

The 5-Fu-loaded hybrid aerogel microspheres were also fabricated via emulsification and chemical cross-linking [17,26,31,32,33]. In a typical procedure, 0, 15, 30, 45 mg GO was dispersed into 1 mL distilled water under ultrasonic. The obtained GO dispersion and 0.3 g CS were added into 15 mL 5% acetic acid aqueous solution, and 0, 0.15, 0.3, or 0.45 g HPC was added to the dispersion under stirring until complete dissolution at 50 °C. Then, 0.1 g 5-Fu was added to the mixture and stirred for an hour; the obtained mixture was poured into an emulsifier dispersion, which consisted of 1.8 mL Tween-80, 49 mL petroleum, and 35 mL liquid paraffin. Half an hour later, 2 mL glutaraldehyde was dropped within 30 min, and the chemical cross-linking reaction was maintained at 60 °C for another hour. Finally, the fabricated GO/HPC/CS@5-Fu aerogel was washed with hexane and water alternately and dried at room temperature for 24 h in a vacuum oven.

### 4.4. Characterization

The surface morphology was observed using scanning electron microscopy (VEGA-3 SBH, Tescan, Czech Republic). Fourier transform infrared (FTIR) spectra were recorded on an Avatar 360 Nicolet instrument (Thermo Fisher Scientific, Shanghai, China) using KBr pellets, in wave numbers ranging from 4000 to 400 cm^−1^ with a resolution of 4 cm^−1^. The crystal structures of the aerogel were investigated by X-ray powder diffraction (LabX XRD-6100, Shimazdu, Japan). The differential scanning calorimeter (DSC) curves were recorded with a DSC-200-F3 (Netzsch, Selb, Germany) from 25 to 400 °C, with a heating rate of 10 °C/min. The thermogravimetry (TG) analysis was conducted via a NETZSCH TG 209F3 instrument (NETZSCH Scientific Instruments, Shanghai, China) under an N_2_ atmosphere with a heating rate of 10 °C/min^−1^.

### 4.5. Calibration Plot of 5-Fu under Different pH Values

**Figure 10:**
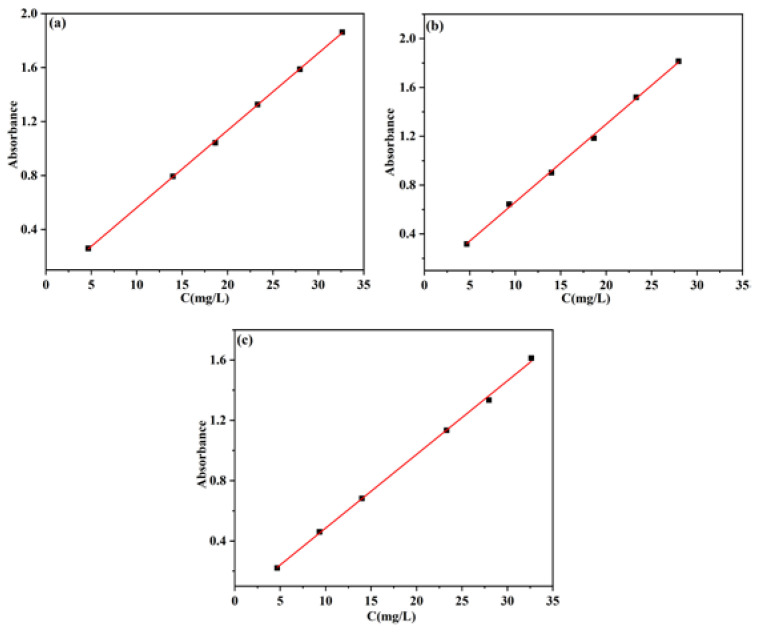
Calibration plot of the absorbance of 5-Fu toward concentration under pH = 1 (**a**), pH = 5.0 (**b**), pH = 7.4 (**c**).

The concentration of 5-Fu in the solution was precisely measured using S 3100 UV–Vis spectra (Mapada Instruments Co. Ltd., Shanghai, China) at 265 nm [27,28]. The determination of drug loading and encapsulation efficiency (pH = 1.0, HCl aqueous solution) and the release rate of 5-Fu (pH = 5.0 and 7.4, PBS solution) were performed at different pH values; therefore, the calibration plot of 5-Fu toward concentration under different pH values was drawn up. The calibration plots are shown in Figure 10, and the corresponding correlation equation and coefficient (*R*^2^) obtained from standard curves are listed in Table 4.

### 4.6. Determination of Drug Loading (DL) and Encapsulation Efficiency (EE)

As reported in the literature [9,24,35], 50 mg of aerogel was accurately weighed and placed in 50 mL HCl aqueous solution (pH = 1) and desorption of 5-Fu occurred at 60 °C for 3 h. Next, the absorbance of 1 mL of filtrate was measured at 265 nm. The drug loading (DL) and encapsulation efficiency (EE) were calculated using the following equation:

DL = [Drug loading/(Drug loading + Aerogel)] *×* 100% (5)

EE = (Determined drug loading/(Theoretic drug loading) *×* 100% (6)

### 4.7. In Vitro Release of 5-Fu from the Aerogel

Typically, the 5-Fu release profiles of HPC/CS@5-Fu and GO/HPC/CS@5-Fu were investigated under two different pH values (5.0 and 7.4) at 37 °C [25,26,27,28]. Briefly, the aerogel samples were accurately weighed and placed into dialysis bags, and the dialysis bags were placed into phosphate-buffered saline (PBS) solution. At specific time intervals during shaking, 3 mL of the PBS was taken out for the determination of the released 5-FU, and an equal volume of fresh PBS was added to the system. The cumulative drug release was calculated based on the following equation:

*Q_n_* = [(*C_n_* × *V*_0_ + *V* × ∑*C*_*n*−1_)/*m*] × 100% (7)

where *m* is the mass of the loaded drug, *C_n_* and *C_n_*_−1_ are the concentration for sampling for *n* times and *n* − 1 times, *V*_0_ and *V* are the initial volume and the sampling volume.

## Figures and Tables

**Table 1:** Kinetic parameters of four models for 5-Fu release at pH = 7.4.

Kinetic Models	Zero-Order		First-Order		Higuchi		Korsmeyer–Peppas		
	*K*_0_	*R* ^2^	*K*_1_	*R* ^2^	*K_H_*	*R* ^2^	*n*	*K_KP_*	*R* ^2^
GO:HPC:CS = 0:3:2	0.01587	0.43968	0.03879	0.63493	0.11106	0.73099	0.25	0.39474	0.93946
GO:HPC:CS = 0.1:3:2	0.01425	0.44393	0.02735	0.60144	0.09934	0.72988	0.30	0.30255	0.92293
GO:HPC:CS = 0.2:3:2	0.01346	0.47198	0.02333	0.6118	0.09295	0.75123	0.33	0.25102	0.91659
GO:HPC:CS = 0.3:3:2	0.01448	0.73457	0.02283	0.83238	0.09311	0.93352	0.32	0.18857	0.93713

**Table 2:** Kinetic parameters of four models for 5-Fu release at pH = 5.0.

Kinetic Models	Zero-Order		First-Order		Higuchi		Korsmeyer–Peppas		
	*K*_0_	*R* ^2^	*K*_1_	*R* ^2^	*K_H_*	*R* ^2^	*n*	*K_KP_*	*R* ^2^
GO:HPC:CS = 0:3:2	0.06865	0.2245	0.47816	0.78216	0.27909	0.49761	0.45	0.58515	0.7224
GO:HPC:CS = 0.1:3:2	0.06748	0.26312	0.26751	0.63888	0.27043	0.53558	0.48	0.52435	0.72266
GO:HPC:CS = 0.2:3:2	0.06155	0.28474	0.15272	0.5382	0.24402	0.55136	0.60	0.39770	0.69554
GO:HPC:CS = 0.3:3:2	0.0613	0.47375	0.12026	0.6588	0.23172	0.73596	0.58	0.31943	0.8017

**Table 3:** Drug carrier materials based on GO and CS biocomposites.

Materials	Drug	Morphology	Loading (%)	Release Time (h)	Total Release (%)	Ref.
CS/HPC/GO	5-Fu	microsphere	89.2	96	98	This work
CS/CMC/GO	5-Fu	aerogel	54.0	10	98	[28]
CS/HPMC	5-Fu	microsphere	59.0	10	98	[17]
CS/MC	5-Fu	nanosphere	99.2	6	100	[16]
CS/NE	5-Fu	nanoparticle	72.4	24	80	[13]
CS/HEC/rGO	FA	nanosheets	92.0	120	40	[23]
CS/GO	MTD	microsphere	36.9	84	90	[25]
CS/GO	AMX	microsphere	89.0	48	40	[26]
CS/GO	DOX	nanoparticle	98.0	50	33	[21]

**Table 4:** Correlation equation and coefficient (*R*^2^) under different pH values.

pH	Equation	*R* ^2^
1.0	*A =* 0.05726 *C −* 0.01049	0.9998
5.0	*A =* 0.06377 *C +* 0.0234	0.9986
7.4	*A =* 0.04887 *C −* 0.00352	0.9987

## Data Availability

Not applicable.

## References

[1] Moutabian H., Majdaeen M., Ghahramani R., Yadollahi M., Gharepapagh E., Ataei G., Falahatpour Z., Bagheri H., Farhood B. (2022). A systematic review of the therapeutic efects of resveratrol in combination with 5-fuorouracil during colorectal cancer treatment: With a special focus on the oxidant, apoptotic, and anti-infammatory activities. Cancer Cell Int..

[2] Safarpour S., Safarpour S., Pirzadeh M., Moghadamnia A.A., Ebrahimpour A., Shirafkan F., Mansoori R., Kazemi S., Hosseini M. (2022). Colchicine ameliorates 5-fluorouracil-induced cardiotoxicity in rats. Oxid. Med. Cell. Longev..

[3] Chen J.Z., Qiu M.J., Zhang S.Q., Li B.B., Li D., Huang X.Y., Qian Z.R., Zhao J., Wang Z.Y., Tang D. (2022). A calcium phosphate drug carrier loading with 5-flfluorouracil achieving a synergistic effect for pancreatic cancer therapy. J. Colloid Interface Sci..

[4] Lopez S., Lopez J.R., Garcia M.T., Rodriguez J.F., Ortiz J.M.P., Ramos M.J., Gracia I. (2022). Self-assembled coumarin- and 5-fluorouracil-PEG micelles as multifunctional drug delivery systems. J. Drug Deliv. Sci. Technol..

[5] Vodenkova S., Buchler T., Cervena K., Veskrnova V., Vodicka P., Vymetalkova Y. (2020). 5-fluorouracil and other fluoropyrimidines in colorectal cancer: Past, present and future. Pharmacol. Therapeut..

[6] Sun Z.Y., Song C.J., Wang C., Hu Y.Q., Wu J.H. (2020). Hydrogel-based controlled drug delivery for cancer treatment: A review. Mol. Pharm..

[7] Webber M.J., Pashuck E.T. (2021). (Macro)molecular self-assembly for hydrogel drug delivery. Adv. Drug Deliv. Rev..

[8] Wu M.Y., Deng W.F., Zhang Y.D., Chen C., Liu Z.X., Fatehi P., Li B. (2022). Facile fabrication of cellulose nanofifibrils/chitosan beads as the potential pH-sensitive drug carriers. Polymers.

[9] Hu Y., Zhang S.W., Han D.D., Ding Z.X., Zeng S.Y., Xiao X.C. (2018). Construction and evaluation of the hydroxypropyl methyl cellulose-sodium alginate composite hydrogel system for sustained drug release. J. Polym. Res..

[10] Bulut E., Turhan Y. (2021). Synthesis and characterization of temperature-sensitive microspheres based on acrylamide grafted hydroxypropyl cellulose and chitosan for the controlled release of amoxicillin trihydrate. Int. J. Biol. Macromol..

[11] Jafari H., Hassanpour M., Akbari A., Rezaie J., Gohari G., Mahdavinia G.R., Jabbari E. (2021). Characterization of pH-sensitive chitosan/hydroxypropyl methylcellulose composite nanoparticles for delivery of melatonin in cancer therapy. Mater. Lett..

[12] Rokhade A.P., Kulkarni P.V., Mallikarjuna N.N., Aminabhavi T.M. (2009). Preparation and characterization of novel semi-interpenetrating polymer network hydrogel microspheres of chitosan and hydroxypropyl cellulose for controlled release of chlorothiazide. J. Microencapsul..

[13] Nawaz A., Latif M.S., Alnuwaiser M.A., Ullah S., Iqbal M., Alfatama M., Lim V. (2022). Synthesis and characterization of chitosan-decorated nanoemulsion gel of 5-fluorouracil for topical delivery. Gels.

[14] Aydin R.S.T., Pulat M. (2012). 5-Fluorouracil encapsulated chitosan nanoparticles for pH-stimulated drug delivery: Evaluation of controlled release kinetics. J. Nanomater..

[15] Bhat S.K., Keshavayya J., Kulkarni V.H., Reddy V.K., Kulkarni P.V., Kulkarni A.R. (2012). Preparation and characterization of crosslinked chitosan microspheres for the colonic delivery of 5-fluorouracil. J. Appl. Polym. Sci..

[16] Sanli O., Kahraman A., Solak E.K., Olukman M. (2015). Preparation of magnetite-chitosan/methylcellulose nanospheres by entrapment and adsorption techniques for targeting the anti-cancer drug 5-fluorouracil. Artif. Cell Nanomed. Biotechnol..

[17] Reddy L.C.N., Reddy R.S., Rao K.K., Subha M., Rao C.K. (2013). Development of polymeric blend microspheres from chitosan-hydroxypropylmethyl cellulose for controlled release of an anti-cancer drug. J. Korean Chem. Soc..

[18] Ji H.W., Sun H.J., Qu X.G. (2016). Antibacterial applications of graphene-based nanomaterials: Recent achievements and challenges. Adv. Drug Deliv. Rev..

[19] Gao J., Bao F., Feng L.L., Shen K.Y., Zhu Q.D., Wang D.F., Chen T., Ma R., Yan C.J. (2011). Functionalized graphene oxide modified polysebacic anhydride as drug carrier for levofloxacin controlled release. RSC Adv..

[20] Ayucitra A., Angkawijaya A.E., Ju Y.H., Gunarto C., Go A.W., Ismadji S. (2021). Graphene oxide-carboxymethyl cellulose hydrogel beads for uptake and release study of doxorubicin. Asia-Pac. J. Chem. Eng..

[21] Wang C., Zhang Z.Q., Chen B.B., Gu L.Q., Li Y., Yu S.W. (2018). Design and evaluation of galactosylated chitosan/graphene oxide nanoparticles as a drug delivery system. J. Colloid Interface Sci..

[22] Rasoulzadehzali M., Namazi H. (2018). Facile preparation of antibacterial chitosan/graphene oxide-Ag bio-nanocomposite hydrogel beads for controlled release of doxorubicin. Int. J. Biol. Macromol..

[23] Mianehrow H., Afshari R., Mazinani S., Sharif F., Abdouss M. (2016). Introducing a highly dispersed reduced graphene oxide nano-biohybrid employing chitosan/hydroxyethyl cellulose for controlled drug delivery. Int. J. Pharm..

[24] Anirudhan T.S., Sekhar V.C., Athira V.S. (2020). Graphene oxide based functionalized chitosan polyelectrolyte nanocomposite for targeted and pH responsive drug delivery. Int. J. Biol. Macromol..

[25] Kumar G., Chaudhary K., Mogha N.K., Kant A., Masram D.T. (2021). Extended release of metronidazole drug using chitosan/graphene oxide bionanocomposite beads as the drug carrier. ACS Omega.

[26] Pooresmaeil M., Asl E.A., Namazi H. (2021). Simple fabrication of biocompatible chitosan/graphene oxide microspheres for pH-controlled amoxicillin delivery. Eur. Polym. J..

[27] Rana V.K., Choi M.C., Kong J.Y., Kim G.Y., Kim M.J., Kim S.H., Mishra S., Singh R.P., Ha C.S. (2011). Synthesis and drug-delivery behavior of chitosan-functionalized graphene oxide hybrid nanosheets. Macromol. Mater. Eng..

[28] Wang R., Shou D., Lv O.Y., Kong Y., Deng L.H., Shen J. (2017). pH-Controlled drug delivery with hybrid aerogel of chitosan, carboxymethyl cellulose and graphene oxide as the carrier. Int. J. Biol. Macromol..

[29] Han X.B., Gao J., Chen Z.Y., Tang X.Q., Zhao Y., Chen T. (2020). Correlation between microstructure and properties of graphene oxide/waterborne polyurethane composites investigated by positron annihilation spectroscopy. RSC Adv..

[30] Li W., Luo T., Yang Y.J., Tan X., Liu L.F. (2015). Formation of controllable hydrophilic/hydrophobic drug delivery systems by electrospinning of vesicles. Langmuir.

[31] Han X.B., Li R., Miao P., Gao J., Hu G., Zhao Y., Chen T. (2022). Design, synthesis and adsorption evaluation of bio-based lignin/chitosan beads for congo red removal. Materials.

[32] Chen T., Zhao Y., Sang Y., Tang M., Hu G., Han X., Gao J., Ma R. (2021). Facile synthesis of magnetic CS-g-SPSS microspheres via electron beam radiation for effiffifficient removal of methylene blue. J. Saudi Chem. Soc..

[33] Isiklan N., Erol U.H. (2020). Design and evaluation of temperature-responsive chitosan/hydroxypropyl cellulose blend nanospheres for sustainable flurbiprofen release. Int. J. Biol. Macromol..

[34] Joy N., Venugopal D., Samavedi S. (2022). Robust strategies to reduce burst and achieve tunable control over extended drug release from uniaxially electrospun composites. Eur. Polym. J..

[35] Li W., Ba H., Huang P., Zheng A.P., Yang X. (2018). Preparation and properties of 5-fluorouracil-loaded chitosan microspheres for the intranasal administration. Drug Res..

